# De Novo Transcriptomic and Metabolomic Analyses Reveal the Ecological Adaptation of High-Altitude *Bombus pyrosoma*

**DOI:** 10.3390/insects11090631

**Published:** 2020-09-14

**Authors:** Yanjie Liu, Huiyue Zhao, Qihua Luo, Yadong Yang, Guangshuo Zhang, Zhiyong Zhou, Muhammad Naeem, Jiandong An

**Affiliations:** 1 Key Laboratory for Insect-Pollinator Biology of the Ministry of Agriculture, Institute of Apicultural Research, Chinese Academy of Agricultural Sciences, Beijing 100093, China; liuyanjie@caas.cn (Y.L.); zhaohuiyue1124@163.com (H.Z.); zhangguangshuo77@126.com (G.Z.); naeem1633@yahoo.com (M.N.); 2 Miyun District Bureau of Landscape and Forestry, Beijing 101500, China; luoqihua0825@163.com (Q.L.); zhouzhiyong198604@163.com (Z.Z.); 3 Institute of Agricultural Resources and Regional Planning, Chinese Academy of Agricultural Sciences, Beijing 100093, China; yangyadong18@163.com; 4 Plant Protection Department, Ghazi University, Dera Ghazi Khan 32200, Pakistan

**Keywords:** bumblebee adaptation, immune signaling, nutrients metabolism, Tibet Plateau, wild pollinator

## Abstract

**Simple Summary:**

Bumblebees are very important pollinators for many wild and agricultural flowers and play significant roles in natural and agricultural ecosystems. The distribution of bumblebees varies between the species; some species occupy narrow areas, while others have a varied distribution from low to high elevation. *Bombus pyrosoma* is one of the bumblebee species with a highly varied geomorphological distribution range in China. To answer why some bumblebee species have a varied distribution, we compared the transcriptomic and metabolomic data of *B. pyrosoma* from the low-altitude North China Plain and the high-altitude Tibet Plateau. The results showed that energy metabolism and innate immunity of the high-altitude *B. pyrosoma* had been enhanced in order to adapt to the extreme environment of hypoxia and low temperature, compared to the low-altitude bumblebees. This study highlights the ecological adaptation of bumblebees distributed from low- to high-altitude conditions.

**Abstract:**

*Bombus pyrosoma* is one of the most abundant bumblebee species in China, with a distribution range of very varied geomorphology and vegetation, which makes it an ideal pollinator species for research into high-altitude adaptation. Here, we sequenced and assembled transcriptomes of *B. pyrosoma* from the low-altitude North China Plain and the high-altitude Tibet Plateau. Subsequent comparative analysis of de novo transcriptomes from the high- and low-altitude groups identified 675 common upregulated genes (DEGs) in the high-altitude *B. pyrosoma*. These genes were enriched in metabolic pathways and corresponded to enzyme activities involved in energy metabolism. Furthermore, according to joint analysis with comparative metabolomics, we suggest that the metabolism of coenzyme A (CoA) and the metabolism and transport of energy resources contribute to the adaptation of high-altitude *B. pyrosoma*. Meanwhile, we found many common upregulated genes enriched in the Toll and immune deficiency (Imd)signaling pathways that act as important immune defenses in insects, and hypoxia and cold temperatures could induce the upregulation of immune genes in insects. Therefore, we suppose that the Toll and Imd signaling pathways also participated in the high-altitude adaptation of *B. pyrosoma*. Like other organisms, we suggest that the high-altitude adaptation of *B. pyrosoma* is controlled by diverse mechanisms.

## 1. Introduction

The Tibet Plateau represents a classic high-altitude environment including hypoxia, cold temperatures and strong ultraviolet rays, which are very harmful to the survival and development of organisms. Fortunately, organisms, including mammals, birds, fish and insects, have evolved some strategies to adapt to high-altitude environments, such as the maintenance of O_2_ homeostasis and optimal oxygenation, which are essential for the survival of all invertebrate and vertebrate species. For example, several species living at high altitudes, including humans, fossorial mammals, diving animals, and birds, have acquired hypoxia tolerance, which enables these organisms to survive under the condition of a limited oxygen supply [1,2,3,4,5]. In humans, genetic evidence has shown that hypoxia response pathways are responsible for hypoxia in the high-altitude regions of Tibet, and these pathways ultimately regulate organic energy balance through the increased delivery and reduced consumption of oxygen [6,7]. Several studies have examined different organisms, i.e., dogs, pikas, Tibetan horses, Tibetan chickens, snub-nosed monkeys and *Drosophila*, to understand their hypoxic adaptabilities [8,9,10,11,12,13]. These studies have confirmed that the high-altitude adaptations of various animals and insects share some common characteristics in terms of the metabolism of oxygen and production of energy, as in the case of *Drosophila* and high-altitude *Homo sapiens*, which share similar genes that respond to hypoxia [8]. However, the regulation of high-altitude adaptations is a very complicated process involving multiple genes and pathways that also shows some differences among different organisms. For example, the genes involved in nutrition metabolism participate in the high-altitude adaptation of the economically important yak [14]. Six genes associated with lung function, angiogenesis, DNA repair, and respiratory cilia movement were shown to be involved in the high-altitude adaptation of snub-nosed monkeys [9]. The calcium signaling pathway is an important target of selection for hypoxic high-altitude adaptation in Tibetan chickens [11].

Bumblebees are very important wild pollinators and play significant roles in agricultural and natural ecosystems. Some bumblebees are capable of inhabiting altitudes over 5000 m and flying across simulated altitudes above 8000 m [15,16,17]. China has the most diverse bumblebee resources in terms of number, species, and distribution range, which covers various geographical environments, including plains, mountains, and plateaus [15,18,19]. *B. pyrosoma* is one of the most abundant and unique endemic species, with a distribution from the low elevations of the North China Plain to the highlands of the Tibet Plateau, covering a wide variety of ecological habitats [19]. Hypoxia and low temperatures can lead to flight deficiency for many insect groups at high elevations [20]. Thus, we suppose that *B. pyrosoma* has developed some strategies to adapt to different habitats, including varied climates and food sources. Compared to their low-altitude counterparts, *B. pyrosoma* that inhabit the Tibet Plateau must overcome hypoxic and cold environments and limited food sources to achieve survival and reproduction. With this hypothesis in mind, we examined the de novo transcriptome and metabolome of *B. pyrosoma* from the Tibet Plateau and the North China Plain to reveal some regulatory pathways or genes involved in the ecological adaptation of high-altitude *B. pyrosoma*.

## 2. Materials and Methods

### 2.1. B. pyrosoma Sample Collection and RNA-Seq

**Figure 1:**
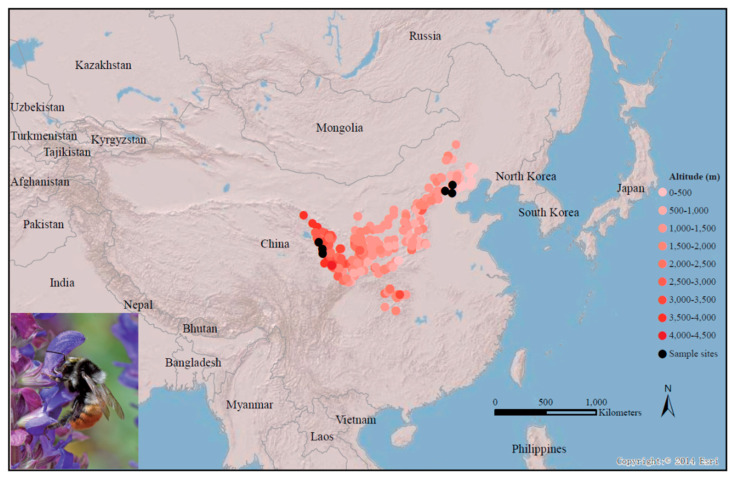
Relief map of China’s mainland showing the recorded distribution (red dots) and the sampling sites (black dots) of *B. pyrosoma* sequenced in this study. The black dots show the three sites (ranging from 522 to 905 m altitude) in the low-altitude North China Plain and the three sites (ranging from 3524 to 3896 m altitude) in the high-altitude Tibet Plateau. The international boundaries are shown in gray, with the names of countries in black. The map was created with ArcGIS v 10.0.

A total of 4927 specimen records of *B. pyrosoma* from our collection showed that *B. pyrosoma* inhabits a very varied geomorphology with an altitude range of 256–3900 m, and 90% of the specimens were distributed from 300–3400 m (Figure 1). Nine worker bees of *B. pyrosoma* were collected from three different sites of the high-altitude Tibet Plateau (103°7′10″ E, 34°15′5″ N, altitude 3599 m, 103°5′6″ E, 34°15′57″ N, altitude 3896 m, 103°7′58″ E, 34°14′52″ N, altitude 3524 m), with each site containing three worker bees as three replicate samples. Another nine worker bees were collected from three different sites of the low-altitude North China Plain (117°28′38″ E, 40°31′56″ N, altitude 905 m, 117°43′44″ E, 41°19′53″ N, altitude 553 m, 115°50′33″ E, 40°28′38″ N, altitude 522 m), with each site containing three worker bees as three replicate samples. All the above samples were foraging bees carrying pollen who shared similar body size and hair colour, which were collected from blooming flowers in July 2019 (the major flying season for worker bees in the study region). Each sample was separated to extract the RNA using TRIzol Reagent (Invitrogen, Karlsbad, CA, USA). RNA degradation and contamination were monitored on 1% agarose gels. RNA purity was checked using the NanoPhotometer^®^ spectrophotometer (Implen, Los Angeles, CA, USA). RNA integrity was assessed using the RNA Nano 6000 Assay Kit on the Agilent Bioanalyzer 2100 system (Agilent Technologies, Palo Alto, CA, USA). A total of 1.5 μg RNA per sample was used as the input material for RNA sample preparation. Sequencing libraries were generated using the NEBNext^®^ Ultra™ RNA Library Prep Kit for Illumina^®^ (New England BioLabs, Ipswich, MA USA) following the manufacturer’s recommendations, and index codes were added to attribute sequences to each sample. Clustering of the index-coded samples was performed on a cBot Cluster Generation System using the TruSeq PE Cluster Kit v3-cBot-HS (Illumia) according to the manufacturer’s instructions. After cluster generation, the library preparations were sequenced on an Illumina Hiseq platform, and paired-end reads were generated. Raw data (raw reads) in the fastq format were first processed through in-house Perl scripts. In this step, clean data (clean reads) were obtained by removing reads containing adapters, reads containing poly-N sequences, and low-quality reads from the raw data. At the same time, the Q20, Q30, GC content and sequence duplication level of the clean data were calculated. All the downstream analyses were based on clean data of high quality.

### 2.2. Transcriptome Assembly and Annotation

Transcriptome assembly was accomplished using Trinity [21] within_kmer_cov set to 2 by default and all other parameters set to the defaults. Then, the transcripts with a length of at least 200 bp were clustered by Corset (https://code.google.com/p/corset-project/) hierarchical clustering to reduce the redundancy. For each cluster, the transcript with the longest region of high-quality sequence data was selected as a gene. Genes were blasted and annotated against the National Center for Biotechnology Information (NCBI) nonredundant protein database (NCBI Nr), NCBI nonredundant nucleotide sequences (NCBI Nt), Protein family (Pfam), clusters of orthologous groups of proteins (KOG/COG), and Swiss-Prot (a manually annotated and reviewed protein sequence database) databases (E-value = <1 × 10^−5^). Genes were blasted to the Nr and Swiss-Prot databases with a priority order, the open reading frames (ORFs) of the blasted genes were extracted, and the coding region sequence was translated into an amino acid sequence according to the standard codon table (in the order of 5′–3′). For genes without blasted results, ORFs were predicted based on estscan (3.0.3) software.

### 2.3. Differentially Expressed Gene Analysis

The expected number of fragments per kilobase of a transcript sequence per million base pairs sequenced (FPKM) is used to evaluate gene expression levels and is the most commonly used method to estimate gene expression levels [22]. Then, compared to the samples from each site of the North China Plain, differential expression analysis of the samples from three sites of the Tibet Plateau was performed using the DEGseq R package [23]. The *p*-values were adjusted using the Benjamini and Hochberg’s approach to control the false-discovery rate [24]. Genes with an adjusted *p*-value <0.05 found by DESeq were assigned as differentially expressed. The common differentially expressed genes in samples from three sites of the Tibet Plateau were examined using the online Veen program (http://bioinformatics.psb.ugent.be/webtools/Venn/).

### 2.4. GO Enrichment and KEGG Enrichment of Common Differentially Expressed Genes

Gene ontology (GO) is a comprehensive description function of the corresponding database (http://www.geneontology.org/), which can divide genes into biological process (in Process), cell (Cellular Component) and molecular functions (Molecular Function). Kyoto Encyclopedia of Genes and Genomes (KEGG) [25] is a database resource for understanding high-level functions and utilities of biological systems, such as the cell, the organism, and the ecosystem, based on molecular-level information, especially for large-scale molecular datasets generated by genome sequencing and other high-throughput experimental technologies (http://www.genome.jp/eg/). We used interproscan [26] software to annotate the GO terms of common upregulated genes and used KOBAS [27] software to test the statistical enrichment of differentially expressed genes in KEGG pathways against the *Apis mellifera* database.

### 2.5. Real-Time Relative Quantitative PCR Verification of Common Differentially Expressed Genes

Eight common differentially expressed genes were randomly chosen and verified by real-time relative quantitative PCR with the bumblebee reference gene actin using a Qtaq™ One-Step qRT-PCR SYBR^®^ KIT (Clontech, Shiga, Japan). All primers are listed in Supplementary Table S3. The real-time PCR data were analyzed using the delta-delta Ct (2^–ΔΔCt^) method [28].

### 2.6. Metabolite Extraction and Metabolomic Analysis of B. pyrosoma Workers

Six *B. pyrosoma* workers of the Tibet Plateau (103°5’6″ E, 34°15’57″ N) and six *B. pyrosoma* workers of the North China Plain (117°28’38″ E, 40°31’56″ N) were collected (the sampling method and criteria were same with the Section 2.1), and each sample was ground with liquid nitrogen. Then, 80 mg of the powder was added to a 1.5 mL tube, and 600 µL cracking solution (MeOH:H_2_O = 1:1) was added. After vortex mixing, the sample was placed overnight in a refrigerator at −20 °C and centrifuged at 4 °C for 20 min at 12,000 rpm. The supernatant was collected, cooled and dried. Then, the residue was combined with 100 µL lysate (MeOH:H_2_O = 1:1) to dissolve the sample into a solution and centrifuged at 4 °C for 20 min at 12,000 rpm. Then, the samples were analyzed by gas chromatography-mass spectrometry (GC-MS). The analytical conditions were a solvent system of water (0.01% formic acid):acetonitrile (0.01% formic acid) and mass data acquisition performed in both positive and negative modes using the turbo spray ion source. To obtain data that could be evaluated for repeatability, quality controls (QCs) were injected periodically throughout the analytical run of the analysis step. Ezinfo software was used for principal component analysis (PCA) and orthogonal Partial Least Squares discriminant analysis (OPLS-DA). OPLS-DA was selected to eliminate the orthogonal signals which were irrelevant to the classification and to screen out the metabolites which caused classification differences. The cross-validation method was used to test the quality of the model (part of the sample data grouping model, which is another part of the data used to test the grouped model). Based on the differential metabolites screened from the samples, the union of the differential metabolites was selected for hierarchical clustering. The KEGG ID values of the unions of different metabolites in the positive and negative ion mode obtained by statistical analysis were input into MetaboAnalyst 4.0, and the different metabolites were compared with the database to obtain the KEGG enrichment analysis.

## 3. Results

### 3.1. RNA-Seq and De Novo Transcriptomic Annotation of B. pyrosoma

RNA-Seq of nine high-altitude samples of *B. pyrosoma* from the three sites of Tibet Plateau and nine low-altitude samples of *B. pyrosoma* from the three sites of North China Plain was conducted. In the first site of the high-altitude group (Hg), 161,737,358 clean reads were generated, and on average, the Q30 value was 92.58%, with a GC content of 42.11% (Table 1). In total, 192,572,982 clean reads were generated from the second site of the high-altitude group, and on average, the Q30 value was 92.81%, with a GC content of 40.81%. A total of 202,292,356 clean reads was generated from the third site of the high-altitude group, and on average, the Q30 value was 92.72%, with a GC content of 41.63%. Overall, 161,258,620 clean reads were generated from the first site of the low-altitude group (Lg), 173,756,628 clean reads were generated from the second site and 173,564,050 clean reads were generated from the third site. The Q30 value was 92.95% at the first site, 92.53% at the second site and 92.89% at the third site of the low-altitude group. The GC contents were 41.24%, 41.18% and 41.95% for the low-altitude group (Table 1). The sequences were deposited into the NCBI Sequence Read Archive (SRA) (accession no. SRR12244814, SRR12245523, SRR12245258, SRR12235348, SRR12233573, SRR12233820).

### 3.2. A Total of 675 Common Upregulated Genes Were Involved in the Adaptation of the High-Altitude B. pyrosoma

**Figure 2:**
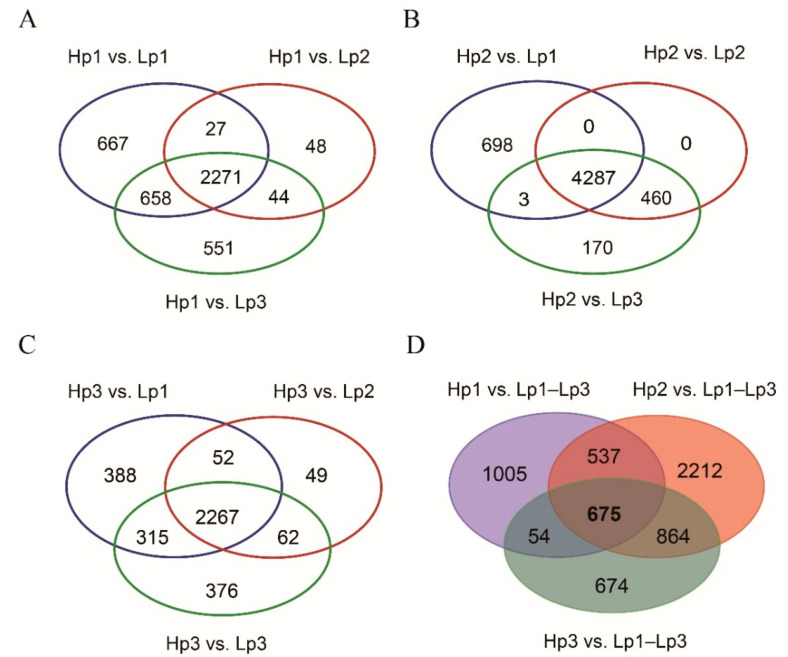
The common upregulated genes of the high-altitude *B. pyrosoma* compared to the low-altitude *B. pyrosoma*. (**A**) The upregulated genes of high-altitude *B. pyrosoma* at the first sampling site compared to the three sites of low-altitude *B. pyrosoma*. (**B**) The upregulated genes of high-altitude *B. pyrosoma* at the second sampling site compared to the three sites of low-altitude *B. pyrosoma*. (**C**) The upregulated genes of high-altitude *B. pyrosoma* at the third sampling site compared to the three sites of low-altitude *B. pyrosoma*. (**D**) The common upregulated genes of high-altitude *B. pyrosoma* among the three sampling sites. Hp represents high-altitude *B. pyrosoma* samples, Lp represents low-altitude *B. pyrosoma* samples, and the Arabic numerals indicate each sampling site.

With a gene expression level cutoff of at least a two-fold difference between the two samples, we identified DEGs between high- and low-altitude *B. pyrosoma*. Compared with the three low-altitude sites, there were 5620, 3889, and 7860 DEGs at the first high-altitude site, among which 3623, 2390, and 3524 genes were upregulated and 1997, 1499, and 4336 genes were downregulated. Compared with the three low-altitude sites, there were 7773, 7479, and 10,862 DEGs at the second high-altitude site, among which 4988, 4747, and 5398 genes were upregulated and 2785, 2732, and 5464 genes were downregulated. Compared with the three low-altitude sites, there were 5246, 4281, and 7502 DEGs at the third high-altitude site, among which 3022, 2430, and 3020 genes were upregulated and 2224, 1851, and 4482 genes were downregulated (Supplementary Figure S1). Among them, the number of common upregulated DEGs was 2271 between the first high-altitude site and the three sites in low-altitude regions (Figure 2A). The number of common upregulated DEGs was 4287 between the second high-altitude site and the three sites in low-altitude regions (Figure 2B). The number of common upregulated DEGs was 2267 between the third high-altitude site and the three sites in low-altitude regions (Figure 2C). In total, there were 675 common upregulated DEGs between the high-altitude and low-altitude regions (Figure 2D and Supplementary Table S1). Correspondingly, the numbers of common downregulated DEGs were 33, 185 and 23, respectively (Supplementary Figure S2A–C). In total, there were only six common downregulated DEGs between the high-altitude and low-altitude regions (Supplementary Figure S2D and Table S1).

### 3.3. GO and KEGG Enrichment of the Common Upregulated Genes

**Figure 3:**
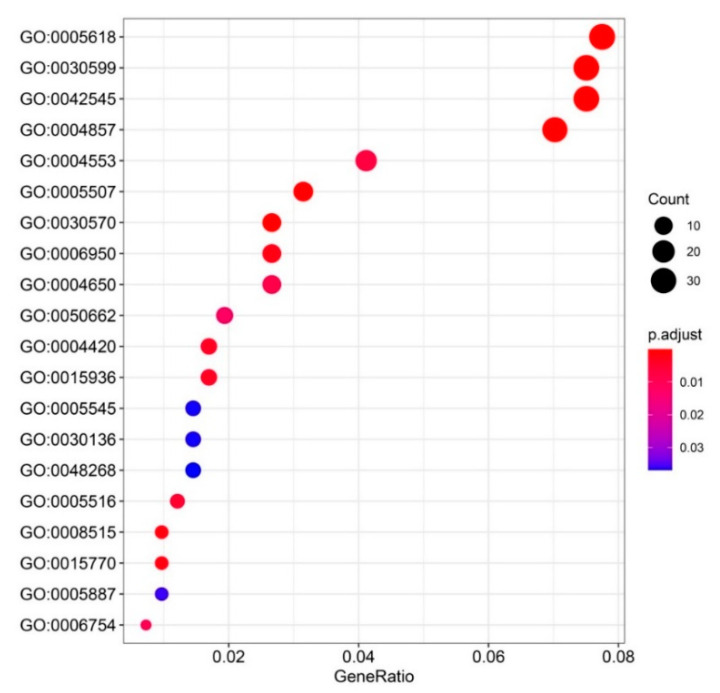
Gene ontology (GO) enrichment of 675 common upregulated genes of the high-altitude *B. pyrosoma* compared to the low-altitude *B. pyrosoma* using interproscan based on the amino acid sequences of common upregulated genes. GO:0005618: cell wall (cell component), GO:0004857: enzyme inhibitor activity, GO:0030599: pectinesterase activity, GO:0042545: cell wall modification, GO:0030570: pectate lyase activity, GO:0005507: copper ion binding, GO:0008515: sucrose transmembrane transporter activity, GO:0015770: sucrose transport (biological process), GO:0006950: response to stress, GO:0004420: hydroxymethylglutaryl-CoA reductase (NADPH) activity, GO:0015936: coenzyme A metabolic process, GO:0005516: calmodulin binding, GO:0004553: hydrolase activity, hydrolyzing *O*-glycosyl compounds, GO:0004650: polygalacturonase activity, GO:0006754: ATP biosynthetic process, GO:0050662: coenzyme binding, GO:0005887: integral component of plasma membrane (cell component), GO:0005545: 1-phosphatidylinositol binding, GO:0030136: clathrin-coated vesicle, GO:0048268: clathrin coat assembly, GO:0016616: oxidoreductase activity, acting on the CH–OH group of donors, with nicotinamide adenine dinucleotide (NAD) or nicotinamide adenine dinucleotide phosphate (NADP) as the acceptor.

**Figure 4:**
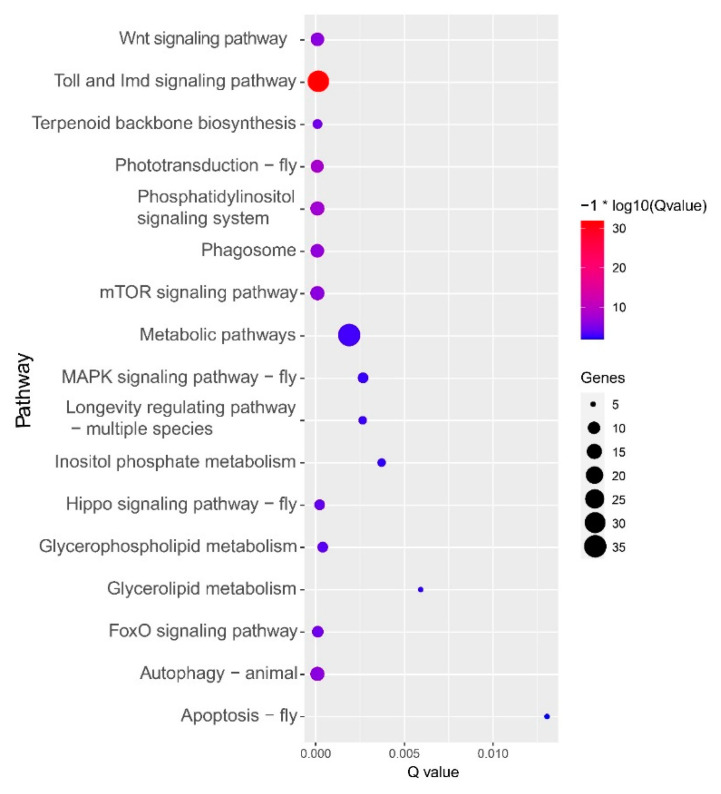
Kyoto Encyclopedia of Genes and Genomes (KEGG) enrichment of common upregulated genes of high-altitude *B. pyrosoma* compared to low-altitude *B. pyrosoma* from three sampling sites using the KOBAS 3.0 server (http://kobas.cbi.pku.edu.cn/) against the *Apis mellifera* database. Imd: immune deficiency, mTOR: mechanistic target of rapamycin, MAPK: mitogen-Activated Protein Kinase.

**Figure 5:**
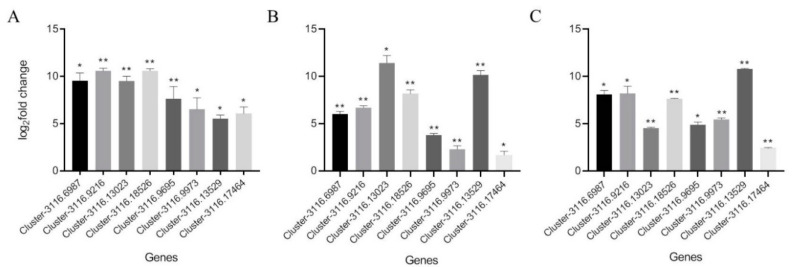
Real-time qPCR results of the eight common upregulated genes, Cluster-3116.6987, Cluster-3116.9216, Cluster-3116.13023, Cluster-3116.18526, Cluster-3116.9695, Cluster-3116.9973, Cluster-3116.13529, Cluster-3116.17464. (**A**) Real-time qPCR results of the above eight common upregulated genes of high-altitude *B. pyrosoma* in comparison with sequenced low-altitude *B. pyrosoma*; (**B**) with low-altitude *B. pyrosoma* (111°19’39″ E, 37°47’6″ N, altitude 1372 m); (**C**) with low-altitude *B. pyrosoma* (109°2’23″ E, 33°47’41″ N, altitude 1116 m). The *X*-axis represents the common upregulated genes in high-altitude *B. pyrosoma*, the *Y*-axis represents the values of relative expression levels, * represents *p*-value < 0.05 and ** represents *p*-value < 0.01.

In total, 22 GO terms were enriched by the common upregulated genes. These GO terms contain cell components, biological processes and molecular functions, and most focus on molecular functions (Figure 3). The biological processes include sucrose transport, the coenzyme A metabolic process, and responses to stress. The molecular functions are mainly associated with enzyme activities, such as enzyme inhibitor activity, pectate lyase activity, pectinesterase activity, sucrose transmembrane transporter activity, hydroxymethylglutaryl-CoA reductase activity, hydrolase activity, polygalacturonase activity, and oxidoreductase activity. In addition, the molecular functions also involve the ATP biosynthetic process and 1-phosphatidylinositol binding. Among the common upregulated genes enriched on 17 KEGG pathways, most were localized on the Toll and Imd signaling pathways and metabolic pathways (Figure 4). Other primary enriched KEGG pathways include the autophagy–animal pathway, wnt signaling pathway, phototransduction–fly, phagosome pathway, phospatidylinositol signaling system, mechanistic target of rapamycin (mTOR) signaling pathway, and FoxO signaling pathway. The randomly selected common upregulated genes have been verified by real-time qPCR in high-altitude *B. pyrosoma* in comparison with sequenced low-altitude *B. pyrosoma* (Figure 5A) and the low-altitude *B. pyrosoma* from other two distant sites (Figure 5B,C). Real-time qPCR data for these selected genes confirmed that they were upregulated in samples collected from over 3000 m altitude, compared to various distant low-altitude samples with a range from 522 to 1372 m.

### 3.4. Metabolic Differences between High- and Low-Altitude B. pyrosoma

**Figure 6:**
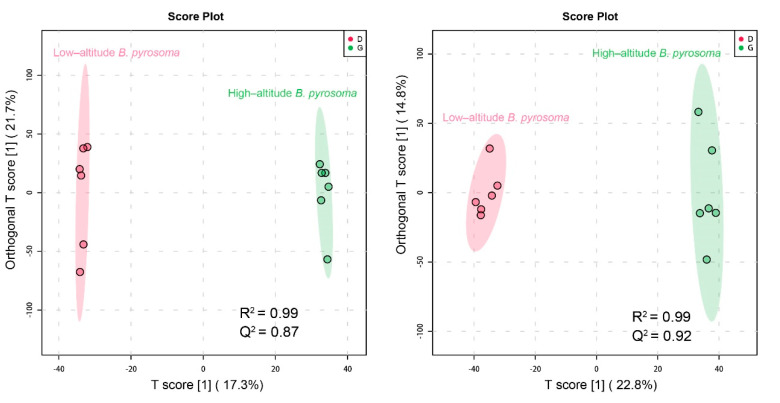
The score plots of orthogonal Partial Least Squares discriminant analysis (OPLS-DA) in negative and positive ion modes of the high-altitude and low-altitude *B. pyrosoma*, respectively, with *n* = 6 per group. D: low-altitude *B. pyrosoma*, G: high-altitude *B. pyrosoma*.

To obtain adequate information concerning metabolism, both positive and negative ion modes were applied to analyze the *B. pyrosoma* samples. In this experiment, 6245 features were detected in the negative ion mode, and 7085 features were detected in the positive ion mode. The obtained R^2^X and Q^2^ respectively represent the explicable variables and the predictability of the model, which can be used to judge the advantages and disadvantages of the model. The R^2^X and Q^2^ of the OPLS-DA in this experiment were 0.99 and 0.92 in negative ion mode and 0.99 and 0.87 in positive ion mode (Figure 6). The data showed that there was no overfitting phenomenon in the model, and the model had a good prediction ability for grouping.

**Figure 7:**
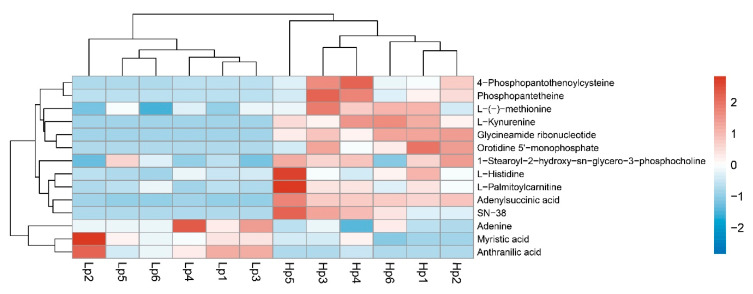
Heatmap of the differential metabolites of high-altitude compared to low-altitude *B. pyrosoma*. Hp represents high-altitude *B. pyrosoma* samples, Lp represents low-altitude *B. pyrosoma* samples, and the Arabic numerals indicate each sample.

In total, 484 differential metabolites were identified (Supplementary Table S2). Compared to the *Drosophila* database, these differential metabolites could be enriched in pantothenate and CoA biosynthesis, histidine metabolism, tryptophan metabolism, β-alanine metabolism, purine metabolism, alanine, aspartate and glutamate metabolism, aminoacyl-tRNA biosynthesis, drug metabolism (other enzymes), glycerophospholipid metabolism, cysteine and methionine metabolism, fatty acid degradation, pyrimidine metabolism, and fatty acid biosynthesis pathways (Table 2). Cluster analysis of the enriched metabolites shows that most are upregulated, including phosphopantetheine, 4-phosphopantothenoylcysteine, l-histidine, l-kynurenine, orotidine 5′-monophosphate, 2′-deoxy-5-hydroxymethyl-CDP, l-(-)-methionine, l-oalmitoylcarnitine, glycineamide ribonucleotide, 1-stearoyl-2-hydroxy-sn-glycero-3-phosphocholine, SN-38 and adenylsuccinic acid (Figure 7). Therefore, we supposed that the upregulated metabolites should be associated with the adaptation of the high-altitude *B. pyrosoma*.

## 4. Discussion

As an important pollinator, *B. pyrosoma* inhabits a wide geomorphology covering plains, mountains and highlands with an altitude range of 256–3900 m in China, which provides good material to study the ecological adaptation of bumblebees [19]. In the current study, the intact de novo transcriptomes of *B. pyrosoma* from the low-altitude region and the high-altitude region were assembled for the first time. We sequenced and assembled *B. pyrosoma* transcriptomes from three sites in the North China Plain and the Tibet plateau, respectively, which made the analysis of differentially expressed genes more precise. Together with the metabolomics analysis, our results showed that the metabolism and transport of energy resources are adaptation responses of high-altitude *B. pyrosoma*. The phenomenon is similar to the reported high-altitude adaptations of other organisms [6,29,30]. Generally, organisms can conserve energy by reducing energy turnover or by increasing energy efficiency under hypoxia. Bumblebees are a particular group of insects that exclusively utilize carbohydrates to power flight, and these insects support flight using sugars from nectar [31]. Carbohydrates from nectar are composed mainly of sucrose and include lesser amounts of fructose and glucose [32]. Among the 675 common upregulated genes in high-altitude *B. pyrosoma*, some are enriched in metabolic pathways and correspond to the biological processes of the coenzyme A metabolic process and sucrose transport, and some are enriched in the molecular functions of sucrose transmembrane transporter activity, ATP biosynthesis, hydrolase activity, and oxidoreductase activity. All these genes are associated with the glycometabolism process and the production of energy, which provide the necessary energy in the normal flight of *B. pyrosoma* under the high-altitude environment. Moreover, the enriched pathways of differential metabolites include the pantothenate and CoA biosynthesis pathways, and the enriched metabolites phosphopantetheine and 4-phosphopantothenoylcysteine were upregulated in high-altitude *B. pyrosoma*. Coenzyme A is a fundamental cofactor in all living organisms and is a highly versatile molecule, serving metabolic functions in both anabolic and catabolic pathways such as fatty acid synthesis, amino acid metabolism and the citric acid cycle [33,34]. Low air densities and cold temperatures limit the flight of insects that possess a powerful respiratory system due to more complications than those caused by hypoxia [35,36,37]. Therefore, under cold temperatures and hypoxic environments, the high-altitude *B. pyrosoma* might accelerate the metabolism of nutrients including sugars, amino acids and fatty acids to provide enough energy to support normal flight. Moreover, we found several metabolites involved in amino acid metabolism and fatty acid metabolism, which partly explains how amino acids and fatty acids might be involved in the ecological adaptation of *B. pyrosoma* in addition to sugars. Furthermore, the enriched pathways of common upregulated genes include the autophagy–animal and mTOR signaling pathways, which are also relevant to the metabolism of amino acids and fatty acids [38,39]. It has been reported that nutrients are mobilized from body fat to support other tissues largely through autophagy, and indeed, autophagy-defective *Drosophila* mutants die more rapidly [40,41]. Body fat plays a major role in the storage and release of energy in response to the energy demands of insects [42]. Autophagy could be inhibited by the mTOR complex in humans [43], which is needed for body homeostasis. Thus, we suggest that autophagy and the mTOR signaling pathway participated in the ecological adaptation of high-altitude *B. pyrosoma* that inhabit nectar- and pollen-scarce geographic areas. In addition, the bumblebees inhabiting the Tibet Plateau possess a shorter period to finish colony development because of the blooming period of wildflowers is shorter in the Tibet Plateau. So, we supposed that the more efficient nutrient metabolisms are able to facilitate high-altitude *B. pyrosoma* to finish colony reproduction under the harsh environment and shorter life cycle. Remarkably, many common upregulated genes enriched in the Toll and Imd signaling pathways act as important immune defenses in insects. The Toll and Imd signaling pathways were activated by microorganisms and regulated the production and release of antimicrobial peptides to eliminate invasion and infection by pathogens [44]. In vitro experiments have shown that insects exposed to the cold have upregulated expression of immune-related genes, including those coding for antimicrobial peptides [45,46]. Moreover, hypoxia could activate IκB kinase (IKK)/NF-κB and the immune response in *D. melanogaster*, which is important to the survival of *D. melanogaster* [47], and activation of the NF-κB pathway in *Drosophila* can occur via the Toll and Imd pathways [48]. Our analysis is in agreement with these previous findings, and we suppose that the Toll and Imd signaling pathways are involved in the regulation of the high-altitude adaptation of bumblebees. However, it is still unclear why high-altitude environments, including hypoxic conditions and cold temperatures, would activate the immune response. Except for the common upregulated genes, the fewer common downregulated genes also might contribute to the survival and development of high-altitude *B. pyrosoma*, which needs further research to verify. This study helps expand our knowledge of the ecological adaptation of bumblebees to the Tibetan environment.

## 5. Conclusions

Our results showed that the ecological adaptation of *B. pyrosoma* involves many different mechanisms and energy metabolism that is very important to the adaptation, which is consistent with previous reporting about the adaptation of other organisms. Moreover, we found that the Toll and Imd immune signaling pathways also take part in the regulatory mechanisms of the ecological adaptation of high-altitude *B. pyrosoma*.

## Figures and Tables

**Table 1:** Summary of RNA-seq quality data of 18 *B. pyrosoma* samples.

Samples	Raw Reads	Clean Reads	Clean Bases	Error Rate	Q20	Q30	GC (%)
Hg1_1	49,658,386	48,916,486	7.34G	0.03	97.12	92.05	41.9
Hg1_2	60,422,470	59,299,792	8.89G	0.03	97.44	92.75	42.01
Hg1_3	54,353,120	53,521,080	8.03G	0.03	97.54	92.95	42.42
Hg2_1	58,759,526	57,894,918	8.68G	0.03	97.62	93.15	40.92
Hg2_2	66,679,540	65,591,808	9.84G	0.03	97.41	92.67	40.95
Hg2_3	70,088,970	69,086,256	10.36G	0.03	97.37	92.62	40.56
Hg3_1	68,686,072	67,882,044	10.18G	0.03	97.3	92.39	41.03
Hg3_2	59,035,522	58,092,164	8.71G	0.03	97.58	93.06	42.19
Hg3_3	77600512	76,318,148	11.45G	0.03	97.45	92.72	41.66
Lg1_1	50,739,698	49,722,722	7.46G	0.03	97.68	93.25	42.08
Lg1_2	65,200,928	64,057,478	9.61G	0.03	97.42	92.67	40.53
Lg1_3	48,444,610	47,478,420	7.12G	0.03	97.52	92.93	41.1
Lg2_1	55,991,666	54,658,022	8.2G	0.03	96.89	91.44	41.03
Lg2_2	62,258,242	61,271,324	9.19G	0.03	97.55	92.97	41.48
Lg2_3	58,823,612	57,827,336	8.67G	0.03	97.67	93.18	41.03
Lg3_1	59,367,598	58,397,304	8.76G	0.03	97.53	92.89	42.24
Lg3_2	55,118,938	54,163,008	8.12G	0.03	97.5	92.85	41.18
Lg3_3	62,453,414	61,003,738	9.15G	0.03	97.54	92.93	42.44

**Table 2:** The statistics of the KEGG enrichment of the differential metabolites.

KEGG Pathways	Total	Hits	*p*-Value	Impact	Metabolites
Pantothenate and CoA biosynthesis	18	2	0.33422	0.47223	Phosphopantetheine; 4-Phosphopantothenoylcysteine
Histidine metabolism	9	1	0.45929	0.4	l-Histidine
Tryptophan metabolism	30	2	0.5996	0.12451	Anthranilic acid; l-Kynurenine
β-alanine metabolism	14	1	0.61666	0	l-Histidine
Pyrimidine metabolism	40	2	0.75488	0.08308	Orotidine 5′-monophosphate; 2′-deoxy-5-hydroxymethyl-CDP
Aminoacyl-tRNA biosynthesis	48	2	0.83941	0	l-Histidine; l-(-)-methionine
Cysteine and methionine metabolism	32	1	0.89043	0.13814	l-(-)-methionine
Fatty acid degradation	38	1	0.92819	0	l-Palmitoylcarnitine
Purine metabolism	63	2	0.93117	0.04192	Glycineamide ribonucleotide; Adenine
Fatty acid biosynthesis	43	1	0.9496	0	Myristic acid
Alanine, aspartate and glutamate metabolism	23	1	0.77956	0.02027	Adenylsuccinic acid
Drug metabolism (other enzymes)	31	1	0.87077	0.02273	SN-38
Glycerophospholipid metabolism	32	1	0.87916	0.04362	1-Stearoyl-2-hydroxy-sn-glycero-3-phosphocholine

## Supplementary Materials

The following are available online at https://www.mdpi.com/2075-4450/11/9/631/s1, Figure S1: The statistics of the differentially expressed genes in high-altitude *B. pyrosoma* compared to the low-altitude *B. pyrosoma*, Figure S2: The common downregulated genes of the high-altitude *B. pyrosoma* compared to the low-altitude *B. pyrosoma*, Table S1: common regulated gene list, Table S2: differential metabolites list, Table S3: Primers of the real-time qPCR verification in this study.

## Abbreviations

Lg	Low-altitude group
Hg	High-altitude group
