# Supplementary material for: De Novo Transcriptomic and Metabolomic Analyses Reveal the Ecological Adaptation of High-Altitude Bombus pyrosoma
Source: Insects. 2020 Sep 14;11(9):631. doi: 10.3390/insects11090631 (PMC7563474; doi:10.3390/insects11090631)
Supplement: Supplementary file 1 [file insects-11-00631-s001.zip › insects-906754-SI/Supplementary figures.docx]

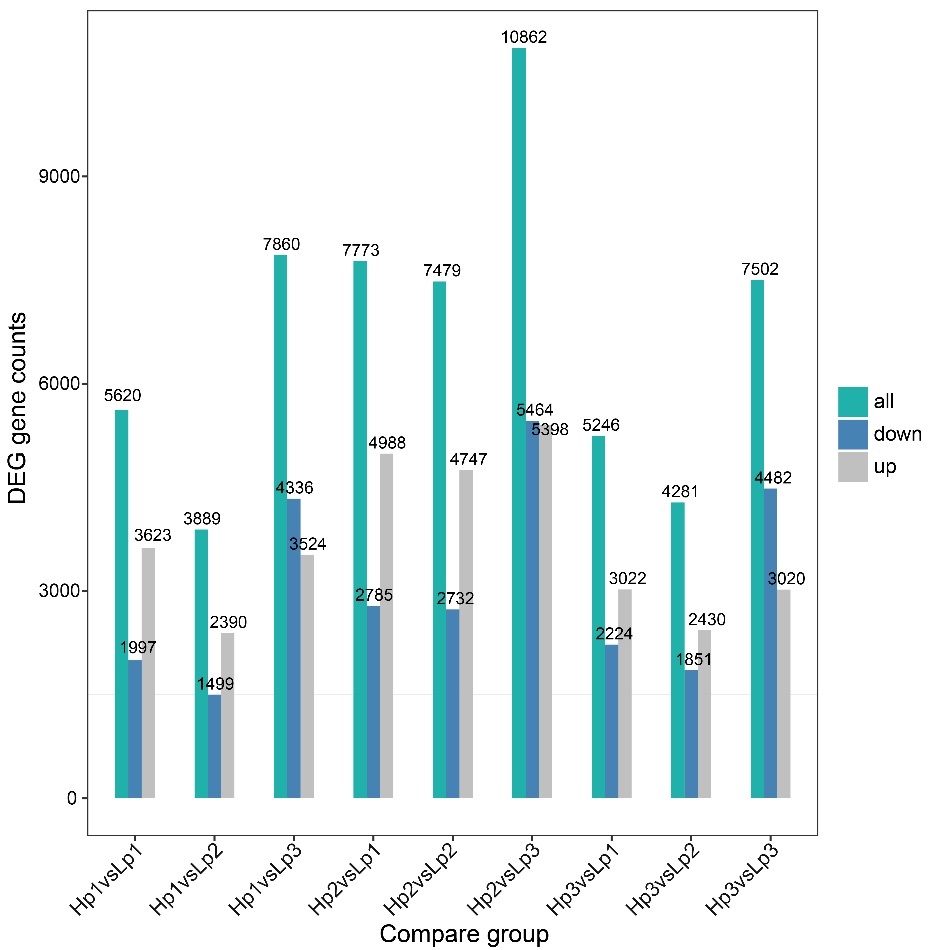


Figure S1. The statistics of the differentially expressed genes in high-altitude *B. pyrosoma* compared to the low-altitude *B. pyrosoma*. Hp represents High-altitude *B. pyrosoma*, Lp represents Low-altitude *B. pyrosoma.*


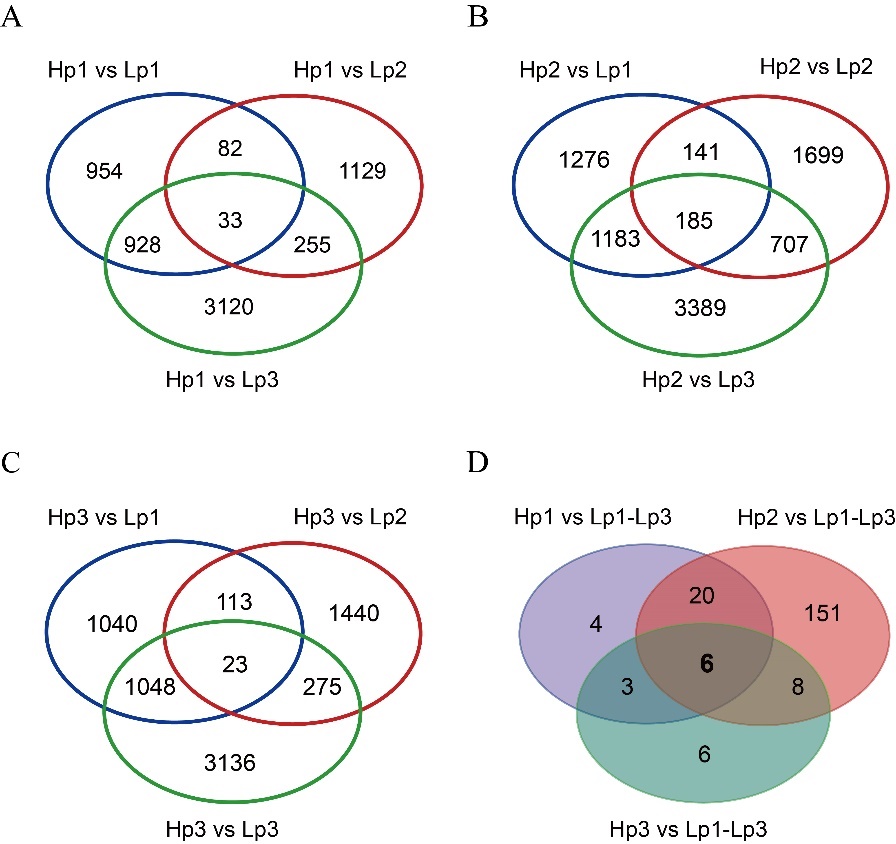


Figure S2. The common downregulated genes of the high-altitude *B. pyrosoma* compared to the low-altitude *B. pyrosoma*. A. The downregulated genes of high-altitude *B. pyrosoma* at the first sampling site compared to the three sites of low-altitude *B. pyrosoma*. B. The downregulated genes of high-altitude *B. pyrosoma* at the second sampling site compared to the three sites of low-altitude *B. pyrosoma*. C. The downregulated genes of high-altitude *B. pyrosoma* at the third sampling site compared to the three sites of low-altitude *B. pyrosoma*. D. The common downregulated genes of high-altitude *B. pyrosoma* among the three sampling sites.
