# Supplementary material for: De Novo Transcriptomic and Metabolomic Analyses Reveal the Ecological Adaptation of High-Altitude Bombus pyrosoma
Source: Insects. 2020 Sep 14;11(9):631. doi: 10.3390/insects11090631 (PMC7563474; doi:10.3390/insects11090631)
Supplement: Supplementary file 1 [file insects-11-00631-s001.zip › insects-906754-SI/Table S3.docx]

**Table S3. Primers of the real-time qPCR verification in this study**

| **Genes ID** | **Primers name** | **Primers sequences** |
| --- | --- | --- |
| **Actin** | α-actin F | ACCTCCCTTGAGAAGAGCTACG |
|  | α-actin R | TACCCAGGAAGGAAGGTTGG |
| **Cluster-3116.6987** | 6987 F | TTTCATCCCCAGCACCTCAC |
|  | 6987 R | ACACAGCCTCACCCTAAACG |
| **Cluster-3116.9216** | 9216 F | TTTGTAGGGGCACGTCCTTC |
|  | 9216 R | CGGTGAAATGGGCAATCGAC |
| **Cluster-3116.9695** | 9695 F | GTGGTGCTACCATGGCTCTT |
|  | 9695 R | TTCGGTGTGGCTGTTGCTAT |
| **Cluster-3116.9973** | 9973 F | TCGCTTCACCGCAACTTTTG |
|  | 9973 R | GCGTACGATGGGTATGGGTT |
| **Cluster-3116.13023** | 13023 F | AACTCCAGGCAGAACGATGG |
|  | 13023 R | CAGCTTTCATAACCGGCAGC |
| **Cluster-3116.13529** | 13529 F | AGGTACGAGGAGATGGTGGAG |
|  | 13529 R | GTTCTTGTAGGCGACGGAGAG |
| **Cluster-3116.17464** | 17464 F | GGCAAGAACGGCGACGACAA |
|  | 17464 R | GCTGTGGGATTGGAACCTGACG |
| **Cluster-3116.18526** | 18526 F | TCGGCTTCTGGAGGGATCAAGG |
|  | 18526 R | GTGCCATAGTGCTGAACGCTGA |
